# When Future Focus Hinders Rest: Temporal Focus, Perceived Involution, and Rest Intolerance Among Chinese University Students

**DOI:** 10.3390/bs16091582

**Published:** 2026-09-07

**Authors:** Kexin Huang, Jiaqin Yang, Chunlei Liu

**Affiliations:** School of Psychology, Qufu Normal University, Qufu 273165, China

**Keywords:** rest intolerance, temporal focus, perceived involution, psychological recovery, motivational conflict

## Abstract

Rest intolerance—the difficulty of truly relaxing during rest, accompanied by guilt and anxiety—is increasingly prevalent among university students, yet its attentional antecedents remain poorly understood. Drawing on motivational conflict theory, this research examined temporal focus as a correlate of rest intolerance and perceived involution as a boundary condition. Study 1, a cross-sectional survey of 290 Chinese university students, found that trait future temporal focus was positively associated with rest intolerance, whereas present temporal focus was negatively associated with it, with perceived involution moderating both associations. An exploratory latent profile analysis identified three profiles—balanced (80.7%), future-focused (10.7%), and present-focused (8.6%)—with the future-focused profile showing the highest rest intolerance. Study 2, a 2 (temporal focus: future vs. present) × 2 (perceived involution: high vs. low) experiment with 131 participants, found that induced future focus was related to higher reported rest intolerance than induced present focus, particularly under low involution; under high involution, this difference was reduced. These findings identify temporal focus as a systematic attentional factor relevant to rest intolerance and suggest the potential applicability of motivational conflict theory to the rest domain.

## 1. Introduction

Rest is a fundamental human need, essential for maintaining physical health and psychological well-being (Sonnentag & Fritz, 2007). High-quality rest enables individuals to detach from work and academic demands, recover depleted cognitive resources, and restore self-regulatory capacity (Zijlstra & Sonnentag, 2006). However, an increasing number of individuals, particularly university students, report experiencing difficulty truly relaxing during rest periods. Instead of rejuvenation, they experience guilt, anxiety, obsessive thoughts about unfinished tasks, and social comparison—phenomena collectively conceptualized as “rest intolerance” (Wang et al., 2025).

### 1.1. Rest Intolerance: Conceptualization and Existing Research

Rest intolerance is a recently conceptualized construct describing individuals’ difficulty achieving psychological recovery during rest periods (Wang et al., 2025). Unlike simple workaholism or overwork, rest intolerance specifically captures the psychological struggle during intended rest periods—when individuals try to relax but cannot fully disengage from achievement-related concerns.

Rest intolerance represents a multidimensional psychological construct encompassing four core features: (1) negative emotions such as guilt and anxiety during rest; (2) obsessive thinking about work or academic tasks; (3) social comparison regarding others’ productivity; and (4) cognitive distortions that devalue rest (Wang et al., 2025). This phenomenon is particularly relevant in Chinese university contexts, where intense academic competition and achievement-oriented social norms create psychological pressure to maximize productive time, and where perceived involution has been systematically associated with students’ subjective well-being (Li, 2025).

This multidimensional conceptualization moves beyond earlier unidimensional measures of “leisure guilt” (Koo, 2023) to capture the complex psychological experience of rest in achievement-oriented contexts.

Rest intolerance has significant negative consequences: it undermines the restorative functions of rest, impairs subsequent performance, and is associated with higher anxiety, lower subjective well-being, reduced actual leisure time, and poorer self-rated health (Avcı, 2026; Koo, 2023; Wang et al., 2025). Understanding its antecedents is therefore essential for developing interventions that promote healthy rest patterns.

Since its recent conceptualization, a small but growing body of research has begun to map the nomological network of rest intolerance. Wang et al. (2025) developed and validated the Rest Intolerance Scale among Chinese college students, establishing its four-dimensional structure and links to academic stress. Subsequent studies have shown that rest intolerance is associated with emotional distress and insomnia (Avcı, 2026), that it is embedded in a network of academic and psychological factors among nursing students (Zhao et al., 2025), and that it prospectively undermines health-promoting behavior such as physical exercise (D. Zhang et al., 2026). What remains largely unexamined, however, is which habitual attentional patterns predispose individuals to rest intolerance—in particular, whether the way students allocate attention across time systematically shapes their ability to recover during rest.

### 1.2. Temporal Focus: Future and Present Orientations

Temporal focus refers to the degree to which individuals habitually direct their attention toward the past, present, or future (Shipp & Aeon, 2019; Shipp et al., 2009). Two dimensions are particularly relevant to rest experiences: future temporal focus (attention to future goals and long-term planning) and present temporal focus (attention to immediate experiences and current activities).

Unlike time perspective, which often carries evaluative connotations, temporal focus is a more neutral construct describing attentional patterns without presupposing their adaptive value.

Future temporal focus involves directing attention toward future events, long-term goals, and anticipated outcomes. Future-focused individuals engage in planning, delay gratification, and prioritize long-term consequences over immediate rewards—characteristics that closely parallel the future dimension of time perspective (Zimbardo & Boyd, 1999). Future temporal focus is generally associated with adaptive outcomes such as greater goal-directed behavior (Shipp & Aeon, 2019; Shipp et al., 2009). While generally adaptive for achievement, excessive future focus may come at the cost of present-moment engagement and enjoyment; more broadly, time perspective research has linked individuals’ temporal orientations to their well-being (Drake et al., 2008).

Present temporal focus involves attending to current experiences, immediate sensations, and ongoing activities. Present-focused individuals are more likely to engage mindfully with their current circumstances and derive satisfaction from immediate experiences (Brown & Ryan, 2003). Related research on temporal perspective shows that how individuals weight the past, present, and future in daily life is systematically related to their well-being (Rush & Grouzet, 2012), and research on mindfulness—conceptually related to present focus—demonstrates that attending to the present moment enhances well-being and reduces psychological distress (Brown & Ryan, 2003).

### 1.3. Theoretical Framework: Motivational Conflict Theory

We adopt motivational conflict theory as our guiding framework for three reasons. First, rest intolerance is at its core an experience of conflict: individuals intend to rest yet feel pulled toward achievement goals. Second, the theory’s central construct—motivational interference, the intrusion of an unchosen but still-activated goal into current activity—offers a precise mechanism for the guilt, rumination, and comparison that define rest intolerance. Third, the theory specifies boundary conditions (goal strength and temporal salience) that allow us to derive testable, person-specific predictions about who is most vulnerable. Throughout this paper, the theory informs both our hypotheses (which attentional patterns should intensify interference) and our interpretation of when rest fails as recovery.

Motivational conflict theory (Lewin, 1931) provides a useful lens for understanding rest intolerance. According to this framework, individuals often pursue multiple attractive but incompatible goals within limited time and resource constraints (Hofer et al., 2009). When individuals choose one goal over another, the unselected goal remains activated and interferes with the current activity—a process termed “motivational interference” (King & Gaerlan, 2013). This interference manifests as attentional distraction, reduced behavioral persistence, and negative emotions such as guilt and anxiety.

Previous research has extensively examined how leisure motivation interferes with academic engagement (Hofer et al., 2009; King & Gaerlan, 2013). However, the reverse direction—how achievement motivation interferes with rest states—has received less attention, particularly among student populations. Rest intolerance can be conceptualized as motivational interference where future-oriented achievement goals intrude upon and disrupt the rest state.

### 1.4. Temporal Focus, Motivational Interference, and Rest Intolerance

Applying this framework to rest contexts, we propose that rest intolerance arises when achievement goals interfere with the rest state. Future-focused individuals may experience stronger motivational interference during rest because strong future orientation can carry a compulsive drive to keep investing time in future goals even at the expense of recovery (Keller et al., 2016). When time is spent on rest rather than productive activities, these individuals may perceive rest as an opportunity cost; the rest state then activates concerns about uncompleted tasks and missed opportunities, producing guilt and anxiety.

Conversely, present temporal focus emphasizes immediate experiences and current needs (Brown & Ryan, 2003). Present-focused individuals may experience weaker motivational interference during rest because they are more likely to evaluate rest based on current psychological and physical needs rather than future productivity, potentially reducing guilt and enabling more complete psychological detachment.

### 1.5. Perceived Involution as a Situational Moderator

Perceived involution refers to individuals’ subjective perception of the involutionary climate within their environment (W. Zhang et al., 2024). In highly involuted academic environments, students perceive that they must continuously outperform peers to secure limited opportunities, creating a “rat race” mentality. Recent evidence indicates that upward social comparison is a central driver of academic involution among college students, exerting both a direct effect and indirect effects through reduced self-esteem and heightened perceived stress (Wen & Jin, 2025).

It encompasses four dimensions: perceived resource scarcity (limited opportunities), social norms (expectations for overwork), psychological pressure (stress and anxiety), and competitive behavior (observed overwork among peers).

Perceived involution may moderate the relationship between temporal focus and rest intolerance. In highly involuted environments, overwork climates elevate the social value of continuous effort (Chen et al., 2026), and rest may be implicitly stigmatized as “falling behind” or “lacking ambition.” For future-focused individuals, high involution amplifies concerns about falling behind, missing opportunities, and competitive disadvantage, strengthening the association between future focus and rest intolerance.

For present-focused individuals, high involution may override their natural tendency to value immediate rest experiences. Even individuals who typically prioritize present needs may feel pressured to conform to competitive norms, weakening the protective effect of present focus on rest intolerance.

Importantly, temporal focus can be conceptualized at two levels: as a relatively stable trait (habitual attentional allocation) and as a momentary state that can be situationally induced. Because Study 1 examines trait-level associations, whereas Study 2 manipulates state temporal focus, the moderating role of perceived involution may operate differently across the two studies. For chronically future-focused individuals, a highly competitive environment continuously supplies salient cues of potential future loss and is therefore expected to *strengthen* the future focus–rest intolerance link (H2). For state-induced focus, however, acute activation of future goals may already be sufficient to render rest an opportunity cost, such that situational pressure exerts its effect primarily by eroding the protection afforded by present focus. We therefore predict that the future-versus-present difference will be most visible under low involution in Study 2 (H5). This trait–state distinction is addressed directly in Section 4.

### 1.6. The Present Research: Research Questions, Two-Study Design, and Hypotheses

The present research addresses three research questions: RQ1: Are trait future and present temporal focus differentially associated with rest intolerance? RQ2: Does perceived involution moderate these associations, and are there distinct temporal focus profiles that differ in rest intolerance? RQ3: Does experimentally induced temporal focus produce corresponding differences in rest intolerance, and does this effect depend on situational involution cues? We present two complementary studies in a single paper because they answer these questions at different levels of analysis: Study 1 establishes trait-level associations and person-centered heterogeneity in a naturalistic sample, while Study 2 tests the causal direction of the key association under controlled conditions. Presenting them together allows the correlational and experimental evidence to mutually constrain interpretation—neither study alone could support the claims we make.

Study 1 uses a cross-sectional survey to examine trait-level associations and identify distinct temporal focus profiles using latent profile analysis. Study 2 employs experimental manipulation to test the causal direction of these associations.

Based on the above theoretical analysis, we propose the following hypotheses:

**H1.**  *Future temporal focus positively predicts rest intolerance, while present temporal focus negatively predicts rest intolerance*.

**H2.**  *Perceived involution moderates the relationships between temporal focus and rest intolerance. Specifically, high perceived involution strengthens the positive association between future temporal focus and rest intolerance and weakens the negative association between present temporal focus and rest intolerance*.

**H3.**  *Distinct temporal focus profiles exist (e.g., future-focused, present-focused, balanced), and these profiles differ significantly in rest intolerance, with future-focused individuals showing the highest levels*.

**H4.**  *Experimentally induced future temporal focus results in higher rest intolerance than induced present temporal focus*.

**H5.**  *Temporal focus and perceived involution interact to influence rest intolerance, with the difference between future- and present-focused conditions being larger under low involution than under high involution*.

## 2. Study 1

### 2.1. Materials and Methods

#### 2.1.1. Participants

A total of 310 university students were initially recruited through the Credamo online survey platform (https://www.credamo.com, accessed on 2 December 2025). Following recommended data-screening procedures, we excluded 20 respondents who failed an embedded attention-check item, exhibited invariant or patterned responding, or completed the survey in an implausibly short time, yielding a final sample of 290 valid cases (194 females, 96 males; Mage = 22.00 years, SD = 2.00). Participants were from various provinces, including Guangdong, Shandong, and Beijing. A sensitivity power analysis indicated that with N = 290, α = 0.05, and 80% power, the hierarchical regression models (up to 8 predictors) could detect incremental effects as small as f^2^ ≈ 0.034, indicating adequate power for the hypothesized main and interaction effects. All participants were naïve to the research purpose and had not participated in similar studies. All participants provided informed consent, and the study was approved by the university ethics committee.

#### 2.1.2. Measures

Temporal Focus: Temporal focus was measured using the Chinese version of the Temporal Focus Scale (Shipp et al., 2009; Zhu et al., 2022). The scale includes 12 items assessing attention to the past, present, and future. For this study, we used the 4-item future focus subscale (e.g., “I think about what my future will be like”) and the 4-item present focus subscale (e.g., “I focus on what is currently happening”). Items were rated on a 7-point scale (1 = never, 7 = always). In this study, Cronbach’s α was 0.88 for future focus and 0.87 for present focus.

Perceived Involution: Perceived involution was measured using the Perceived Involution Scale (W. Zhang et al., 2024), which assesses four dimensions: resource scarcity, social norms, psychological pressure, and competitive behavior. The 18-item scale uses a 7-point rating scale (1 = strongly disagree, 7 = strongly agree). Example items include “I feel that resources and opportunities around me are very limited.” Cronbach’s α in this study was 0.75.

Rest Intolerance: Rest intolerance was measured using the 8-item short form of the Rest Intolerance Scale (Wang et al., 2025; see Appendix A). The scale assesses four dimensions: negative emotions, obsessive thinking, social comparison, and cognitive distortions. Items are rated on a 5-point scale (1 = strongly disagree, 5 = strongly agree). Example items include “When resting or entertaining, I worry about being surpassed by others.” Cronbach’s α in this study was 0.91.

Control Variables: We controlled for generalized anxiety (measured by GAD-7, α = 0.89; Spitzer et al., 2006) and subjective socioeconomic status (assessed with the family and school ladders of the MacArthur Scale of Subjective Social Status; Goodman et al., 2001), as these variables have been associated with both temporal focus and rest-related experiences.

#### 2.1.3. Procedure

Participants completed an online questionnaire including all measures. The order of measures was randomized to reduce order effects. After completing the survey, participants were debriefed and compensated.

### 2.2. Results

#### 2.2.1. Preliminary Analyses

Because all variables were assessed via self-report at a single time point, we evaluated common method bias using two approaches. First, Harman’s single-factor test showed that the unrotated exploratory factor analysis of all items from the focal measures extracted eight factors with eigenvalues greater than 1, with the first factor explaining only 25% of the variance—below the 40% threshold. Second, confirmatory factor analyses comparing a single-factor model with the hypothesized four-factor model showed that the single-factor model fit the data poorly (for parcel-level models: χ^2^(77) = 880.6, CFI = 0.59, RMSEA = 0.190) and significantly worse than the four-factor model (χ^2^(71) = 176.6, CFI = 0.95, TLI = 0.93, RMSEA = 0.072; Δχ^2^(6) = 704.0, *p* < 0.001). These results suggest that common method variance is unlikely to account for the observed associations.

Descriptive statistics and correlations among key variables are presented in Table 1. Future temporal focus was positively correlated with rest intolerance (r = 0.56, *p* < 0.01) and perceived involution (r = 0.29, *p* < 0.01). Present temporal focus was negatively correlated with rest intolerance (r = −0.43, *p* < 0.01) and perceived involution (r = −0.15, *p* < 0.05). Perceived involution was positively correlated with rest intolerance (r = 0.57, *p* < 0.01).

#### 2.2.2. Regression Analyses

Hierarchical regression analyses were conducted to test H1 and H2. In Step 1, control variables (generalized anxiety and subjective socioeconomic status) were entered. In Step 2, future temporal focus, present temporal focus, and perceived involution were entered. In Step 3, the interaction terms (Future Focus × Involution, Present Focus × Involution) were entered.

Results (Table 2) showed that after controlling for anxiety and socioeconomic status, future temporal focus positively predicted rest intolerance (β = 0.30, *p* < 0.001), and present temporal focus negatively predicted rest intolerance (β = −0.24, *p* < 0.001), supporting H1. The interaction terms were significant: Future Focus × Involution (β = 0.12, *p* < 0.001) and Present Focus × Involution (β = 0.11, *p* < 0.01), supporting H2.

Simple slopes analyses revealed that at low perceived involution (−1 SD), future temporal focus showed a significant positive association with rest intolerance (b = 0.19, 95% CI [0.09, 0.30], *p* < 0.001). At high perceived involution (+1 SD), this positive association was stronger (b = 0.43, 95% CI [0.31, 0.55], *p* < 0.001).

**Figure 1:**
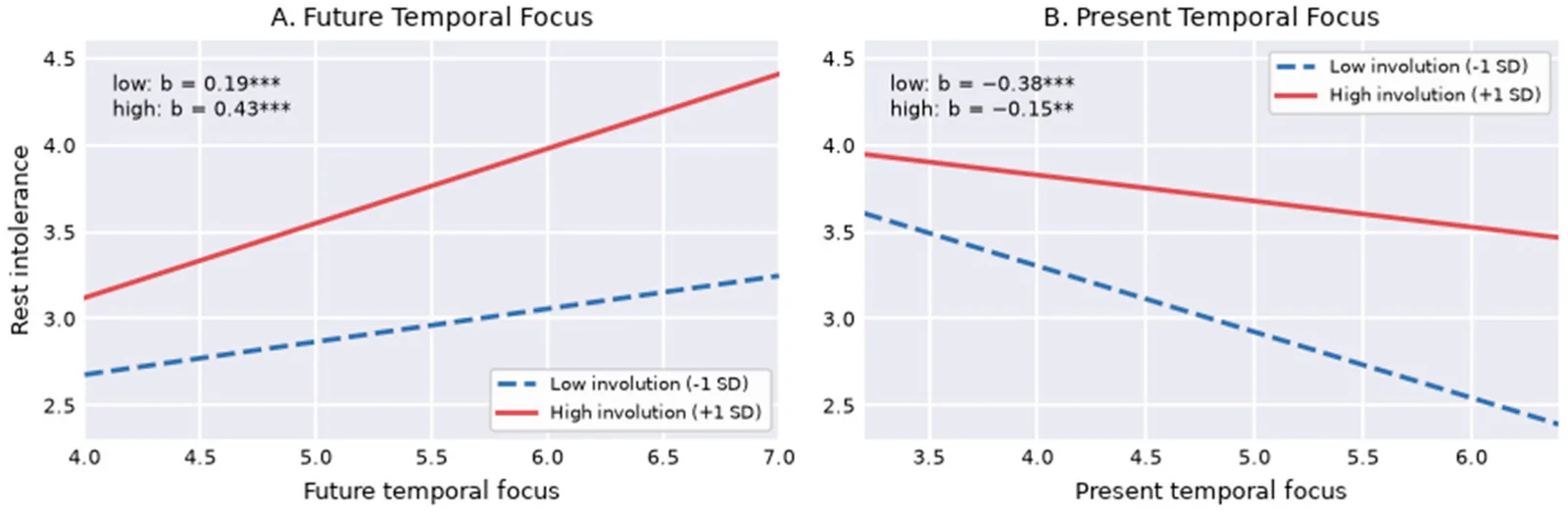
Simple slopes of temporal focus predicting rest intolerance at high and low perceived involution (Study 1). ** *p* < 0.01, *** *p* < 0.001.

For present temporal focus, at low perceived involution, there was a significant negative association with rest intolerance (b = −0.38, 95% CI [−0.51, −0.25], *p* < 0.001). At high perceived involution, this negative association was attenuated (b = −0.15, 95% CI [−0.25, −0.04], *p* < 0.01). The simple slopes for both moderation effects are depicted in Figure 1.

#### 2.2.3. Latent Profile Analysis

Given that the LPA relies on two indicator dimensions, we treat the resulting profiles as exploratory, heuristic descriptions of attentional patterns rather than definitive latent subpopulations. To identify distinct temporal focus profiles and test H3, we conducted latent profile analysis (LPA) using the tidyLPA package (Rosenberg et al., 2018) in R (version 4.4.1; R Core Team, 2024), with the mean scores of the future-focus and present-focus subscales as two continuous indicators. Variances were constrained to be equal across profiles, and covariances were fixed to zero (EEI). Models with one to four profiles were estimated and compared using fit indices (AIC, BIC, and aBIC), entropy, and the Bootstrap Likelihood Ratio Test (BLRT), together with minimum profile size and interpretability.

Fit indices did not unanimously favor a single solution (Table 3). The BIC reached its minimum at three profiles (1564), whereas the AIC and aBIC continued to decrease slightly at four profiles, and entropy declined from 0.91 (two profiles) to 0.82 (three) and 0.71 (four), indicating deteriorating classification quality. The BLRT was significant for each successive model (all *p*s < 0.01). We selected the three-profile solution because (a) it minimized the BIC, which performs best among these indices in simulation studies of indicator-based LPA (Nylund et al., 2007); (b) the four-profile solution split off a small additional profile (approximately 5% of the sample) that differed from an existing profile in level rather than in shape, adding little interpretive value; and (c) its lower entropy (0.71) indicated less reliable classification. Average posterior probabilities for the three-profile solution ranged from 0.85 to 0.94, supporting classification adequacy. We note that with only two indicators, these solutions should be interpreted as a parsimonious summary of the bivariate distribution of future and present focus rather than as finely differentiated latent subpopulations.

**Figure 2:**
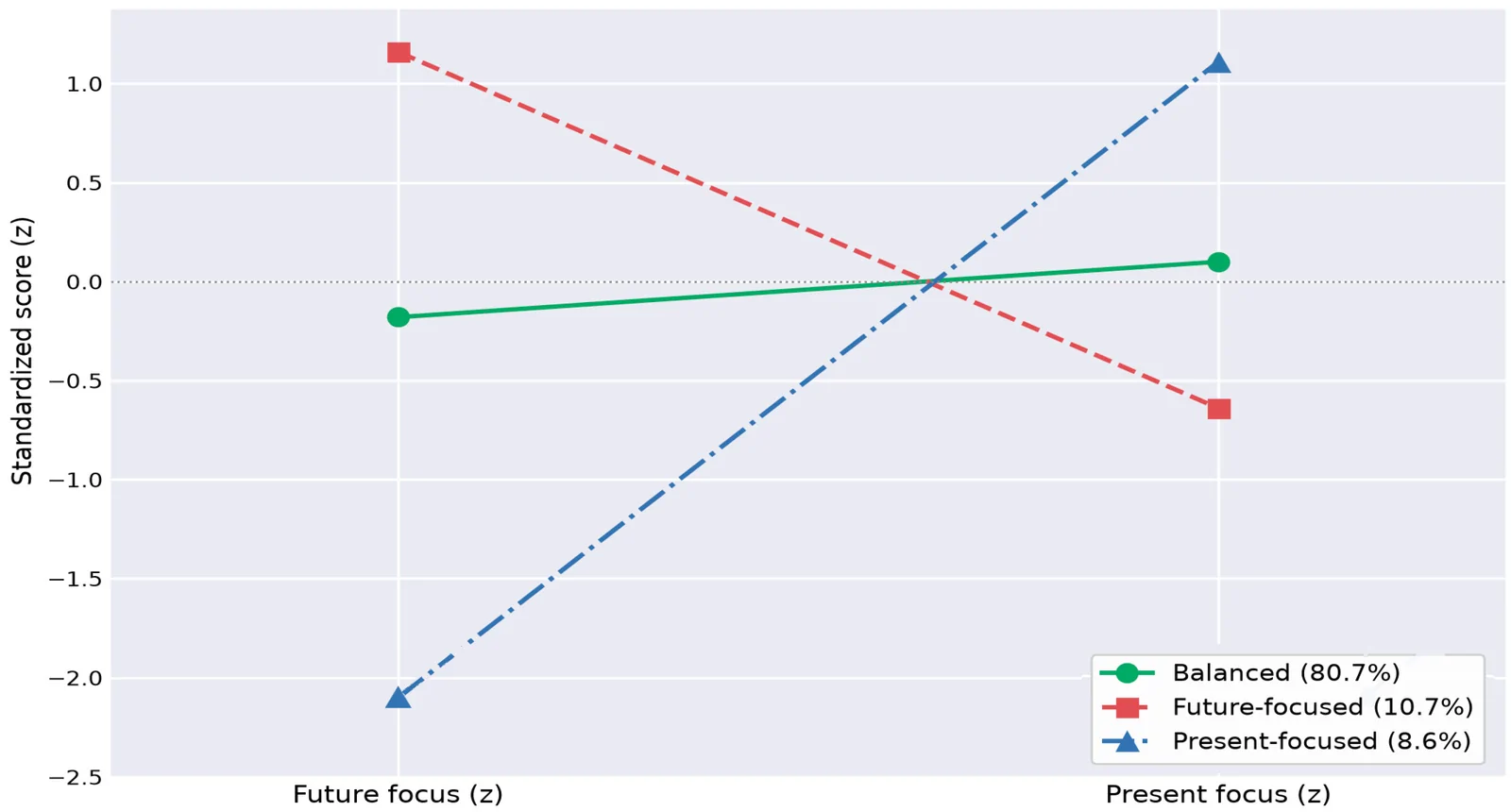
Standardized temporal focus scores for the three latent profiles (Study 1).

The three profiles were labeled as follows: (1) Balanced Type (80.7%)—moderate levels of both future and present focus; (2) Future-Focused Type (10.7%)—high future focus, low present focus; and (3) Present-Focused Type (8.6%)—high present focus, low future focus. The standardized means of the three profiles are shown in Figure 2.

One-way ANOVA revealed significant differences in rest intolerance across profiles (F(2, 287) = 49.45, *p* < 0.001, η_p_^2^ = 0.26). Post hoc Tukey HSD tests showed that future-focused individuals had significantly higher rest intolerance (M = 3.93, SD = 0.72) than balanced individuals (M = 3.43, SD = 0.80), *p* = 0.001, and present-focused individuals (M = 1.98, SD = 0.64), *p* < 0.001. Present-focused individuals had significantly lower rest intolerance than balanced individuals (*p* < 0.001). These findings are consistent with H3.

### 2.3. Brief Discussion

Study 1 provided initial support for the hypothesized relationships. Future temporal focus was positively associated with rest intolerance, while present temporal focus was negatively associated. Perceived involution moderated these relationships: the association between future focus and rest intolerance was stronger under high involution, whereas the negative association between present focus and rest intolerance was attenuated.

The exploratory LPA results suggested that most individuals (80.7%) maintain a balanced temporal focus, while smaller proportions are distinctly future-focused (10.7%) or present-focused (8.6%). Future-focused individuals showed the highest rest intolerance, suggesting that excessive attention to future goals may come at the cost of restorative rest experiences.

However, Study 1’s cross-sectional design limits causal inferences. Study 2 addresses this limitation through experimental manipulation of temporal focus and perceived involution.

## 3. Study 2

### 3.1. Materials and Methods

#### 3.1.1. Participants

A priori power analysis using G*Power 3.1 (Faul et al., 2007) indicated that a minimum sample size of 128 was needed to detect a medium effect size (f = 0.25) with 80% power at α = 0.05 for a 2 × 2 ANOVA. We recruited 136 university students; 5 were excluded for completing the study in less than 3 min, leaving a final sample of 131 (86 females, 45 males; Mage = 21.58, SD = 1.62).

#### 3.1.2. Design and Procedure

The study employed a 2 (temporal focus: future vs. present) × 2 (perceived involution: high vs. low) between-subjects design. Participants were randomly assigned to one of four experimental conditions.

The experiment was administered in a quiet university laboratory setting. Participants were recruited on campus and completed the study individually on computers. Upon arrival, participants provided informed consent and were randomly assigned to one of the four conditions via the survey software’s randomization function. The temporal focus writing task was presented first, followed by the involution scenario materials; participants then completed the dependent measure, manipulation checks, and demographic items in a fixed order. An experimenter was present throughout to answer procedural questions. Sessions lasted approximately 15–20 min, and participants received 5 RMB upon completion.

Temporal Focus Manipulation: Temporal focus was manipulated using a writing task adapted from Dreves and Blackhart (2019). In the future-focused condition, participants read, “Psychologists have found that planning for the future can make life more meaningful and valuable. Many meaningful experiences require long-term planning, such as learning new skills or achieving goals. Please describe your future goals, why they are important, and what actions you will take to achieve them.”

In the present-focused condition, participants read, “Psychologists have found that living in the present can make life more meaningful and valuable. Rather than worrying about the future, people who focus on what happens each day may be happier. Please describe things that make you feel ‘immersed’ in the moment, what you specifically do, and how you feel.” Participants wrote for a minimum of 2 min before proceeding.

Perceived Involution Manipulation: Perceived involution was manipulated using adapted scenario materials (W. Zhang et al., 2024). In the high-involution condition, participants read descriptions emphasizing intense academic competition, limited scholarship opportunities, and peer comparison pressure. Example: “Only students with high GPAs and competition awards receive more recognition and resources.” Participants then wrote about similar experiences.

In the low-involution condition, participants read descriptions emphasizing balanced lifestyles, personal time, and diverse opportunities. Example: “Although there is academic competition, university life overall offers plenty of personal time.” Participants then wrote about similar experiences.

Dependent Variable: Rest intolerance was measured using the same 8-item scale as in Study 1 (Wang et al., 2025; α = 0.91). Because no validated state measure of rest intolerance exists, we used the established trait scale as the outcome; we return to the interpretive constraints of this choice in Section 4.5.

Manipulation Checks: Temporal focus was assessed with a single item: “To what extent are you currently focused on the present versus the future?” (0 = completely focused on the present, 10 = completely focused on the future; Sjåstad, 2019). Perceived involution was assessed with four items adapted from W. Zhang et al. (2024; α = 0.64 in the present study; this value falls below conventional reliability thresholds and should therefore be interpreted with caution, although low internal consistency is not uncommon for brief adapted measures). The single bipolar check item was chosen because state manipulations of temporal focus are brief and the two foci are moderately negatively related at the trait level (r = −0.37 in Study 1); prior experimental work has used a similar single-item bipolar state check (Sjåstad, 2019).

### 3.2. Results

#### 3.2.1. Manipulation Checks

Independent-samples *t*-tests confirmed successful manipulation of both variables. Future-focused participants reported greater future orientation (M = 6.55, SD = 2.34) than present-focused participants (M = 4.13, SD = 2.69), *t*(129) = 5.52, *p* < 0.001, d = 0.96. High-involution participants reported higher perceived involution (M = 4.95, SD = 0.99) than low-involution participants (M = 4.48, SD = 0.90), *t*(129) = 2.86, *p* < 0.01, d = 0.50. In addition, generalized anxiety and subjective socioeconomic status (family and school), assessed as potential covariates, did not differ across the four conditions (Fs(3, 127) ≤ 1.80, *p*s > 0.15), indicating successful randomization.

#### 3.2.2. Main Analyses

A 2 × 2 ANOVA on rest intolerance revealed a significant main effect of temporal focus, F(1, 127) = 15.51, *p* < 0.001, η_p_^2^ = 0.109. Future-focused participants reported higher rest intolerance (M = 3.58, SE = 0.10) than present-focused participants (M = 3.02, SE = 0.10), supporting H4.

**Figure 3:**
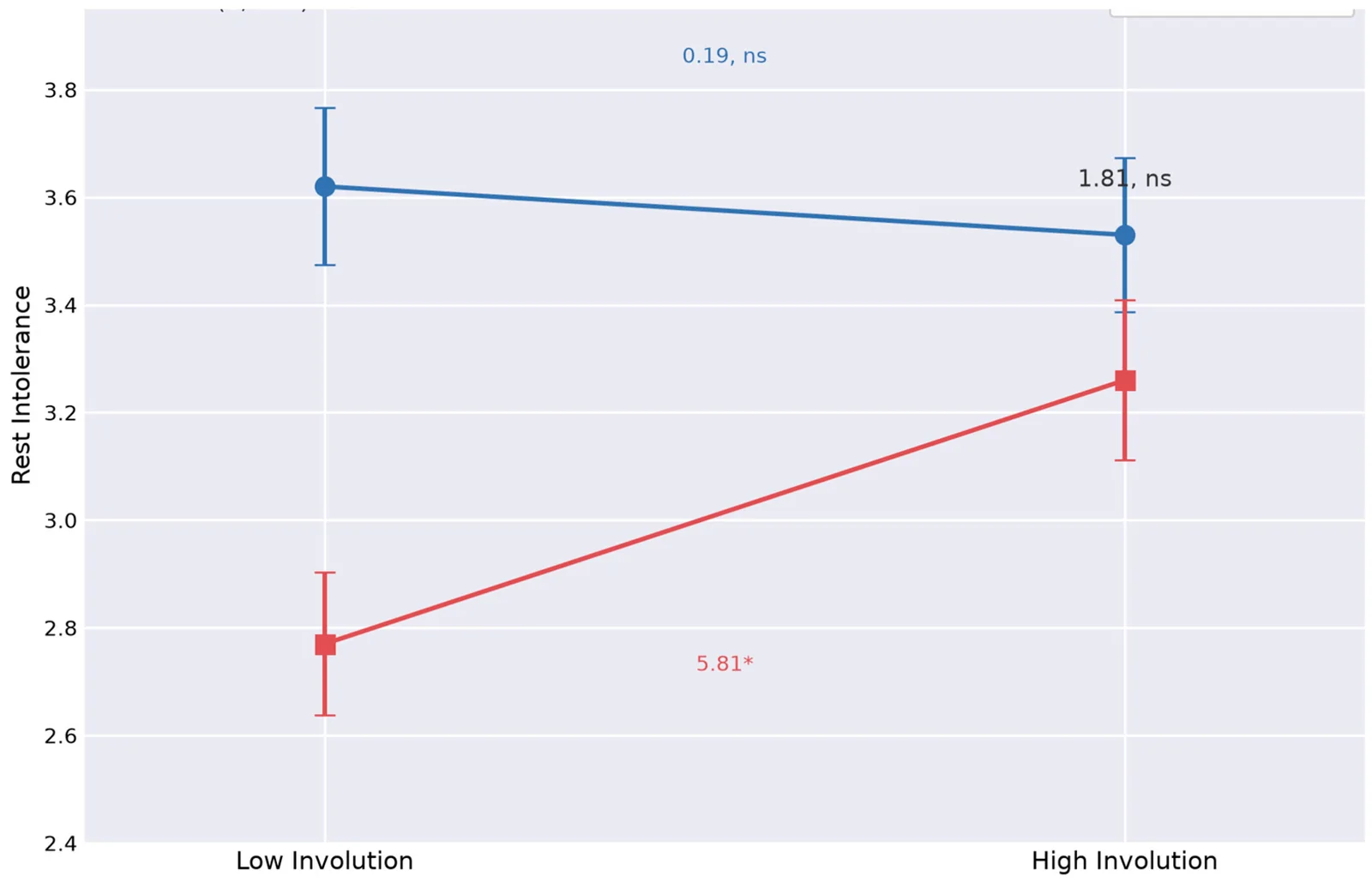
Rest intolerance as a function of temporal focus and perceived involution (Study 2). Error bars represent ±1 SE. The blue and red lines denote the future-focus and present-focus conditions, respectively. * *p* < 0.05; ns = not significant.

The main effect of perceived involution was not significant, F(1, 127) = 2.01, *p* = 0.158, η_p_^2^ = 0.016. Importantly, the interaction between temporal focus and perceived involution was significant, F(1, 127) = 4.12, *p* = 0.044, η_p_^2^ = 0.031, supporting H5. Simple effects analysis revealed that under low involution, future-focused participants showed significantly higher rest intolerance (M = 3.62, SE = 0.14) than present-focused participants (M = 2.77, SE = 0.14), F(1, 127) = 17.94, *p* < 0.001, η_p_^2^ = 0.124. Under high involution, the difference between future focus (M = 3.53, SE = 0.14) and present focus (M = 3.26, SE = 0.14) was not significant, F(1, 127) = 1.81, *p* = 0.181, η_p_^2^ = 0.014. Condition means and standard deviations are reported in Table 4, and the interaction pattern is illustrated in Figure 3.

To decompose the interaction, we further compared involution levels within each temporal focus condition. Among present-focused participants, rest intolerance was significantly higher under high involution (M = 3.26, SD = 0.84) than under low involution (M = 2.77, SD = 0.75), F(1, 127) = 5.81, *p* = 0.017, η_p_^2^ = 0.044. Among future-focused participants, rest intolerance did not differ between high involution (M = 3.53, SD = 0.82) and low involution (M = 3.62, SD = 0.85), F(1, 127) = 0.19, *p* = 0.663, η_p_^2^ = 0.002.

### 3.3. Brief Discussion

Study 2 provided experimental evidence consistent with an effect of state temporal focus on self-reported rest intolerance. More specifically, because the outcome was a trait-oriented measure administered immediately after the manipulation, the causal interpretation is restricted to participants’ scores on that measure in the experimental context and does not license direct inferences about actual rest behavior or chronically experienced rest intolerance. Experimentally induced future temporal focus resulted in higher rest intolerance than induced present temporal focus, replicating and extending Study 1’s correlational findings. The significant interaction revealed that the effect of temporal focus was moderated by perceived involution. Under low involution, future-focused participants showed substantially higher rest intolerance than present-focused participants. However, under high involution, this difference diminished because present-focused participants’ rest intolerance increased significantly, F(1, 127) = 5.81, *p* = 0.017, η_p_^2^ = 0.044. This suggests that high-involution environments can override the protective effect of present temporal focus.

Notably, future-focused participants showed consistently high rest intolerance regardless of involution level: rest intolerance did not differ between the high- and low-involution conditions, F(1, 127) = 0.19, *p* = 0.663, η_p_^2^ = 0.002, a pattern consistent with a ceiling effect. Once future focus is activated, additional situational pressure may have limited incremental impact on rest intolerance. However, this null comparison had limited statistical power and should be interpreted as descriptive rather than as evidence of no effect.

## 4. General Discussion

### 4.1. Summary of Findings

This research examined how temporal focus influences rest intolerance and how perceived involution moderates this relationship through two complementary studies. Study 1 showed that trait future temporal focus positively predicted rest intolerance, while present temporal focus negatively predicted rest intolerance. Perceived involution moderated both relationships, amplifying the association between future focus and rest intolerance and attenuating the protective effect of present focus. Exploratory latent profile analysis identified three distinct temporal focus profiles, with future-focused individuals showing the highest rest intolerance.

Study 2 provided experimental evidence consistent with an effect of state temporal focus on self-reported rest intolerance, with the causal interpretation restricted to scores on the trait-oriented measure administered immediately after the manipulation. Under this constraint, induced future temporal focus was related to higher reported rest intolerance than induced present temporal focus. The interaction between temporal focus and perceived involution revealed that under low involution, future-focused participants showed significantly higher rest intolerance than present-focused participants; this difference disappeared under high involution as present-focused participants’ rest intolerance increased.

### 4.2. Theoretical Implications

This research advances the emerging literature on rest intolerance in three respects. First, it identifies temporal focus as a systematic attentional antecedent of rest intolerance and perceived involution as a boundary condition of this association, thereby extending the nomological network of rest intolerance beyond its established correlates (Wang et al., 2025; Zhao et al., 2025). Second, it suggests the potential applicability of motivational conflict theory to the rest domain: because motivational interference itself was not directly measured, our results are consistent with—rather than a direct test of—the theory. Third, it integrates individual differences (temporal focus) with situational factors (perceived involution), underscoring the value of person–environment fit perspectives for understanding rest-related psychological experiences.

In addition, this research contributes to the temporal focus literature by suggesting that the adaptive value of temporal focus is context-dependent. While future focus is generally associated with positive outcomes such as goal achievement (Shipp & Aeon, 2019; Shipp et al., 2009), it becomes a risk factor in rest contexts. Conversely, present focus, often associated with impulsivity in achievement contexts, serves a protective function during rest. This context-dependent perspective calls into question the assumption that future focus is universally adaptive and present focus is universally maladaptive.

### 4.3. Reconciling the Divergent Moderation Patterns Across Studies

An unexpected but theoretically informative finding emerged: perceived involution moderated the temporal focus–rest intolerance link in opposite directions across the two studies. In Study 1, trait future focus became a stronger risk factor as perceived involution increased, whereas in Study 2, experimentally induced future focus elevated rest intolerance regardless of involution level, and involution primarily weakened the protective effect of present focus.

One plausible account of this divergence is the distinction between chronic and acute future focus. Chronically future-focused individuals habitually monitor future opportunities and threats; a highly competitive environment continuously feeds this monitoring system with salient cues of potential loss, thereby intensifying motivational interference during rest. In contrast, acutely induced future focus may already saturate motivational interference—once future goals are experimentally activated, rest is construed as opportunity cost regardless of ambient competitive pressure, producing a pattern consistent descriptively with a ceiling effect, although this interpretation remains speculative given the limited power for the within-condition contrast. For present-focused individuals, the protective mechanism depends on the environment ‘permitting’ rest: high involution implicitly stigmatizes rest as falling behind, overriding their natural tendency to value immediate recovery. This account aligns with person–environment fit perspectives: the adaptive value of a temporal orientation is jointly determined by its chronicity and the affordances of the situation. This account was not directly tested in the present studies and should be regarded as a hypothesis for future research that manipulates trait and state temporal focus orthogonally—for example, by examining whether trait future focus moderates the effect of state manipulations, or by comparing short-term versus sustained exposure to competitive environments.

### 4.4. Practical Implications

It is important to emphasize that the following implications were not directly evaluated in the present studies; they are offered strictly as directions for future intervention research rather than as empirically verified strategies for promoting healthy rest patterns among students. First, interventions aimed at reducing rest intolerance might incorporate techniques to shift attention toward the present moment during rest periods. Mindfulness-based approaches, which train individuals to attend to present-moment experiences, may be particularly effective (Brown & Ryan, 2003).

Second, reducing perceived involution may help protect rest quality. Educational institutions could consider creating environments that emphasize diverse definitions of success, reduce excessive competition, and normalize rest as essential for long-term performance rather than a sign of laziness. Relatedly, interventions may target the social-comparison processes that fuel involution perceptions—for example, by reducing the salience of upward comparison and strengthening self-esteem and stress-management resources (Wen & Jin, 2025).

Third, individuals high in future temporal focus may benefit from structured rest periods that include explicit permission to disengage from achievement goals. Setting boundaries around work time and rest time, and creating physical or psychological distance from competitive environments during rest, may help future-focused individuals achieve more restorative rest. Each of these proposed strategies awaits direct evaluation in future intervention studies before it can be recommended for practice.

### 4.5. Limitations and Future Directions

Several limitations should be acknowledged.

First and most important for the causal claims of Study 2, rest intolerance was assessed with trait-oriented items immediately after the experimental manipulations. Although the writing tasks were designed to shift participants’ momentary temporal orientation, the measure captured habitual rest experiences, and responses may partly reflect demand characteristics. Future studies should employ state-adapted items (e.g., anticipated experiences during an imminent rest period) or behavioral proxies of rest engagement.

Second, Study 2 manipulated state temporal focus rather than trait temporal focus. While this approach allowed causal inferences, the duration and intensity of the manipulation may not fully capture the long-term effects of chronic temporal focus patterns. Future research could employ longitudinal designs to examine how trait temporal focus develops and influences rest intolerance over time.

Third, both studies relied on self-report measures of rest intolerance. Future research could incorporate behavioral measures (e.g., actual rest duration, physiological indicators of recovery) or experience-sampling methods to capture rest experiences in real time.

Fourth, the samples were limited to Chinese university students. Cultural factors may influence both temporal focus patterns and attitudes toward rest. Future research should examine whether these findings generalize to other cultural contexts and populations (e.g., working adults, non-Asian cultures).

Fifth, the experimental manipulation of perceived involution used written scenarios, which may have limited ecological validity. Future research could examine rest intolerance in actual high- versus low-involution environments or use more immersive manipulations.

Sixth, the moderation patterns differed between trait (Study 1) and state (Study 2) operationalizations of temporal focus. Although we offer a theoretical account, the two studies were not designed to test this divergence directly, and it should be examined in future research with designs that orthogonally manipulate both trait and state temporal focus.

Furthermore, the two-indicator LPA offers limited information for distinguishing profiles; the resulting groups should be regarded as exploratory and replicated with richer indicator sets (e.g., item-level or parcel-level indicators) before strong typological claims are made.

Finally, this research focused on the negative consequences of future focus in rest contexts. Future research could explore whether there are optimal levels of future focus that promote both achievement and well-being, or whether certain strategies (e.g., scheduled future planning time) can satisfy future-focused needs without interfering with rest.

## 5. Conclusions

This research investigated the relationships between temporal focus, perceived involution, and rest intolerance through a cross-sectional survey and an experimental study. Trait future temporal focus was associated with higher rest intolerance, and present temporal focus with lower rest intolerance, with perceived involution moderating these relationships; experimentally induced future focus was related to higher reported rest intolerance than induced present focus. These results call into question the assumption that future focus is universally adaptive and underscore the potential importance of present-moment engagement for restorative rest. Rather than prescribing specific interventions, these findings point to the potential value—for future intervention research—of fostering balanced temporal attention and reducing excessive involution perceptions.

## Figures and Tables

**Table 1:** Means, standard deviations, and correlations among study variables (N = 290).

Variable	M	SD	1	2	3
1. Future Temporal Focus	5.53	0.93	—		
2. Present Temporal Focus	4.81	1.00	−0.37 ***	—	
3. Perceived Involution	4.59	0.54	0.29 ***	−0.15 *	—
4. Rest Intolerance	3.36	0.90	0.56 ***	−0.43 ***	0.57 ***

Note: * *p* < 0.05, *** *p* < 0.001.

**Table 2:** Hierarchical regression analyses predicting rest intolerance (N = 290).

Variable	Step 1	Step 2	Step 3
Control Variables			
Generalized Anxiety	0.49 ***	0.16 **	0.14 *
SES (Family)	−0.06	0.02	0.03
SES (School)	−0.10	0.04	0.05
Main Effects			
Future Temporal Focus		0.30 ***	0.29 ***
Present Temporal Focus		−0.24 ***	−0.25 ***
Perceived Involution		0.34 ***	0.33 ***
Interaction Effects			
Future × Involution			0.12 ***
Present × Involution			0.11 **
ΔR^2^	0.25 ***	0.34 ***	0.02 **
Total R^2^	0.25 ***	0.59 ***	0.61 ***

Note: * *p* < 0.05, ** *p* < 0.01, *** *p* < 0.001. Values are standardized regression coefficients.

**Table 3:** Fit indices for latent profile analysis models.

Profiles	AIC	BIC	aBIC	BLRT (*p*)	Entropy
1	1652	1667	1654	—	—
2	1559	1585	1563	<0.01	0.91
3	1527	1564	1532	<0.01	0.82
4	1520	1568	1527	<0.01	0.71

**Table 4:** Means and standard deviations for rest intolerance by condition.

Condition	n	M	SD
Future Focus/High Involution	33	3.53	0.82
Future Focus/Low Involution	34	3.62	0.85
Present Focus/High Involution	32	3.26	0.84
Present Focus/Low Involution	32	2.77	0.75

## Data Availability

The data that support the findings of this study are available from the corresponding author upon reasonable request. The data are not publicly available due to privacy and ethical restrictions.

## Appendix A

### Appendix A.1. Rest Intolerance Scale (Short Form; Wang et al., 2025)

Instructions: Please rate how much you agree with each statement regarding your experiences during rest or leisure time. (1 = Strongly Disagree to 5 = Strongly Agree)

1. When resting or entertaining, I worry about being surpassed by others.
2. When resting, I often think about peers who are working harder than me.
3. I believe I should spend more time on study/work rather than rest.
4. I think I should use my rest time to do something more meaningful.
5. When resting or entertaining, I feel there are other things I should be doing.
6. When resting or entertaining, I feel guilty.
7. When resting or entertaining, I cannot help thinking about study or work.
8. When resting or entertaining, I feel bad about myself.

### Appendix A.2. Temporal Focus Scale (Future and Present Subscales; Shipp et al., 2009)

Instructions: Please indicate how frequently you engage in each of the following activities. (1 = Never to 7 = Always).

#### Appendix A.2.1. Future Focus Subscale

1. I think about what my future will be like.
2. I focus on my future.
3. I think about what tomorrow will bring.
4. I think about times to come.

#### Appendix A.2.2. Present Focus Subscale

1. I focus on what is currently happening.
2. I focus on the here and now.
3. I live my life in the present.
4. I take each day as it comes.
